# Evolution of Outbreak-Causing Carbapenem-Resistant Klebsiella pneumoniae ST258 at a Tertiary Care Hospital over 8 Years

**DOI:** 10.1128/mBio.01945-19

**Published:** 2019-09-03

**Authors:** Jane W. Marsh, Mustapha M. Mustapha, Marissa P. Griffith, Daniel R. Evans, Chinelo Ezeonwuka, A. William Pasculle, Kathleen A. Shutt, Alexander Sundermann, Ashley M. Ayres, Ryan K. Shields, Ahmed Babiker, Vaughn S. Cooper, Daria Van Tyne, Lee H. Harrison

**Affiliations:** a Infectious Diseases Epidemiology Research Unit, University of Pittsburgh School of Medicine and Graduate School of Public Health, Pittsburgh, Pennsylvania, USA; b Division of Infectious Diseases, University of Pittsburgh School of Medicine, Pittsburgh, Pennsylvania, USA; c Division of Microbiology, University of Pittsburgh Medical Center, Pittsburgh, Pennsylvania, USA; d Division of Hospital Epidemiology and Infection Control, University of Pittsburgh Medical Center, Pittsburgh, Pennsylvania, USA; e Department of Microbiology and Molecular Genetics, University of Pittsburgh School of Medicine, Pittsburgh, Pennsylvania, USA; Louis Stokes Veterans Affairs Medical Center

**Keywords:** carbapenem-resistant, evolution, genomics, molecular epidemiology, outbreaks

## Abstract

Carbapenem-resistant Klebsiella pneumoniae (CRKP) strains belonging to sequence type 258 (ST258) are frequent causes of hospital-associated outbreaks and are a major contributor to the spread of carbapenemases. This genetic lineage emerged several decades ago and remains a major global health care challenge. In this study, genomic epidemiology was used to investigate the emergence, evolution, and persistence of ST258 carbapenem-resistant K. pneumoniae outbreak-causing lineages at a large tertiary care hospital over 8 years. A time-based phylogenetic analysis of 136 ST258 isolates demonstrated the succession of multiple genetically distinct ST258 sublineages over the 8-year period. Ongoing genomic surveillance identified the emergence and persistence of several distinct clonal ST258 populations. Patterns of multidrug resistance determinants and plasmid replicons were consistent with continued evolution and persistence of these populations. Five ST258 outbreaks were documented, including three that were caused by the same clonal lineage. Mutations in genes encoding effectors of biofilm production and iron acquisition were identified among persistent clones. Two emergent lineages bearing K. pneumoniae integrative conjugative element 10 (ICE*Kp10*) and harboring yersiniabactin and colibactin virulence factors were identified. The results show how distinct ST258 subpopulations have evolved and persisted within the same hospital over nearly a decade.

## Abstract (precis)

The carbapenem class of antibiotics is invaluable for the treatment of selected multidrug-resistant Gram-negative pathogens. The continued transmission of carbapenem-resistant bacteria such as ST258 K. pneumoniae is of serious global public health concern, as treatment options for these infections are limited. This genomic epidemiologic investigation traced the natural history of ST258 K. pneumoniae in a single health care setting over nearly a decade. We found that distinct ST258 subpopulations have caused both device-associated and ward-associated outbreaks, and some of these populations remain endemic within our hospital to the present day. The finding of virulence determinants among emergent ST258 clones supports the idea of convergent evolution of drug-resistant and virulent CRKP strains and highlights the need for continued surveillance, prevention, and control efforts to address emergent and evolving ST258 populations in the health care setting.

## INTRODUCTION

Carbapenem-resistant Klebsiella pneumoniae (CRKP) strains belonging to sequence type 258 (ST258) have spread globally in the past several decades. This genetic lineage has caused numerous hospital-associated outbreaks and is of public health concern due to the presence of plasmid-encoded K. pneumoniae carbapenemases (KPCs) (1–6). KPC enzymes hydrolyze all β-lactam antibiotics and limit therapeutic choices for infections caused by multidrug-resistant *Enterobacteriaceae*. Horizontal transfer and recombination among KPC-encoding plasmids occur readily in the hospital environment, contributing to the persistence of CRKP ST258 and the spread of KPCs to other hospital pathogens (7–9). Moreover, the presence of additional antibiotic resistance determinants on KPC plasmids permits dissemination of carbapenemases even in the absence of carbapenem selection (9). Due to its global distribution and epidemic nature and its ability to rapidly disseminate multiple antibiotic resistance determinants, ST258 has been classified as a “high-risk” CRKP lineage (9).

ST258 strains are characteristically divided into two discrete clades on the basis of distinct forms of capsular and plasmid gene content (10). Clade I ST258 isolates typically carry plasmid-encoded KPC-2, while clade II ST258 genomes harbor plasmid-encoded KPC-3. Several recent studies from our institution have documented the development of resistance to colistin therapy and ceftazidime-avibactam therapy among clade I and clade II ST258 strains, respectively (11–14). Additionally, we have previously described multiple device-associated ST258 outbreaks which further highlight the threat posed by this lineage in health care settings (2, 15). The aim of this study was to investigate the emergence, evolution, and persistence of ST258 lineages and their propensity for causing outbreaks in our hospital by studying the genomic epidemiology of a collection of 136 ST258 CRKP isolates collected over an 8-year period.

## RESULTS

### Core genome phylogenetic analyses.

**FIG 1:**
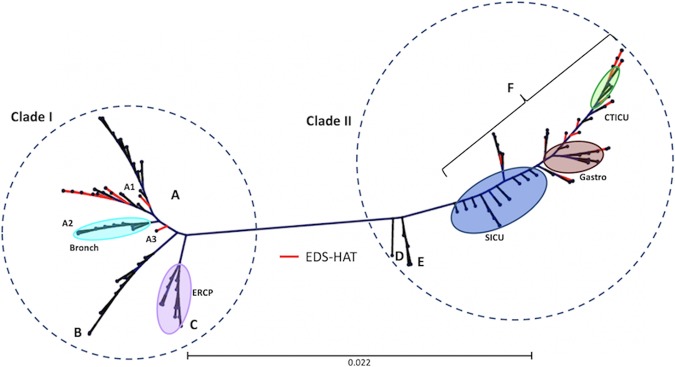
Core genome SNP phylogeny of 136 ST258 isolates collected at UPMC from 2010 to 2018. Dotted circles indicate the two major clades (clade I, *n* =71; clade II = 65). Colored ovals show outbreak-associated isolates with color coding as follows: turquoise, bronchoscope; lavender, ERCP; blue, SICU; brown, gastroscope; green, CTICU; red, EDS-HAT contemporary surveillance isolates. Letters and bracket depict subclades and sublineages described in the text. The scale bar represents the number of substitutions per site.

Whole-genome sequencing (WGS) performed on clinical K. pneumoniae isolates collected for outbreak investigations (*n* = 53), routine infection prevention (IP) (*n* = 17), studies of antimicrobial resistance (AMR) (*n* = 37), and a prospective genomic epidemiology surveillance project, Enhanced Detection System for Hospital-Associated Transmission (EDS-HAT) (*n* = 29), identified 136 isolates belonging to ST258 (see Materials and Methods; see also Table S1 in the supplemental material). A linear relationship between genome length and gene number was observed, validating the quality of the genome assemblies (see Fig. S1A in the supplemental material). A maximum likelihood phylogenetic tree constructed on the basis of 1,130 core genome single nucleotide polymorphisms (cgSNPs) from these genomes was consistent with the ST258 population structure originally described by DeLeo et al. (10) (Fig. 1). Two genetically distinct populations, clade I (*n* = 71) and clade II (*n* = 65), were observed. Clade I isolates harbored the KPC-2 carbapenemase gene and the *wzi*-29 capsular synthesis allele. All clade II isolates carried the *wzi*-154 capsular synthesis allele, and most of the isolates harbored the KPC-3 carbapenemase gene; KPC-3 allelic variants associated with ceftazidime-avibactam resistance were observed in several isolates (Table S1). The two ST258 clades differed from each other by an average of 364 cgSNPs, with a range of 296 to 444 cgSNP differences between pairs of isolates from different clades. The median number of cgSNP differences for isolates within clade I was 64 (range, 0 to 155) and was higher than that for isolates within clade II, which had a median number of cgSNP differences of 16 (range, 0 to 140).

10.1128/mBio.01945-19.1 FIG S1 (A) Scatter plot of genome size versus gene number for each of 136 ST258 K. pneumoniae genomes. (B) Temporal signal among ST258 genomes from isolates collected in 2010 to 2018. Regression analysis of tip-to-root divergence against time using TempEst (17) demonstrated that *R*^2^ = 0.270. Download FIG S1, PDF file, 0.1 MB. Copyright © 2019 Marsh et al. 2019 Marsh et al. This content is distributed under the terms of the Creative Commons Attribution 4.0 International license.

10.1128/mBio.01945-19.6 TABLE S1 Description of the 136 ST258 CRKP isolates used in this study. Download Table S1, XLSX file, 0.02 MB. Copyright © 2019 Marsh et al. 2019 Marsh et al. This content is distributed under the terms of the Creative Commons Attribution 4.0 International license.

Core genome phylogenetic analysis further divided the clade I isolates into three subclades (subclades A, B, and C) (Fig. 1). Isolates belonging to subclade A were the most prevalent (44/71, 62.0%) and comprised three branches on the phylogeny tree (Fig. 1, branches A1 to A3). The A1 branch, which consisted of 32 isolates from 23 patients that were collected between 2010 and 2018, had a median of 27 cgSNP differences (range, 0 to 67) between isolate pairs, suggesting continued transmission and persistence of the A1 clone over the 8-year period. Branch A2 comprised isolates from a 2014 bronchoscopy-associated outbreak, which had a median of 1 cgSNP difference between isolate pairs (range, 0 to 19) (15). Branch A3 comprised a single EDS-HAT isolate collected in 2017. Subclade B isolates (12/71, 16.9%) collected from five patients between 2011 and 2013 had a median of 20 cgSNPs between isolate pairs (range, 0 to 78) (Fig. 1, branch B). Similarly to the A1 isolates, the low number of cgSNPs differences observed among subclade B isolate pairs suggested transmission of this subclade between 2011 and 2013. Subclade C isolates were associated with an endoscopic retrograde cholangiopancreatography (ERCP) outbreak that occurred in 2012 to 2013 and were highly genetically related, with a median of 6 cgSNPs between isolate pairs (range, 0 to 14) (Fig. 1, branch C) (2). These data illustrate the ST258 clade I population structure during the study period and highlight transmission of multiple clade I clones in our hospital as well as the emergence of a persistent lineage (clade I, subclade A).

Three clade II subclades (subclades D, E, and F) were identified in the core genome phylogeny (Fig. 1). A single AMR study isolate, KLP268, belonged to subclade D, and the complete genome sequence of this strain served as the reference for this study (Table S1). Subclade E consisted of six isolates from five patients that were collected in 2010 (10) and that had a median of 13 cgSNPs between isolate pairs (range, 0 to 35). The majority (58/65, 89.2%) of clade II isolates, including 32 isolates from three known outbreaks, belonged to subclade F (Fig. 1; see also Table S1). Thirteen isolates collected between March and August of 2015 belonged to an outbreak in the surgical intensive care unit (SICU) (Fig. 1, branch F, blue oval) (16). A gastroscope-associated outbreak that included 9 isolates occurred in January of 2016, and 10 isolates from a ward-based cluster collected from the cardiothoracic intensive care unit (CTICU) were collected from September to November of the same year (Fig. 1, branch F, brown and green ovals, respectively). In addition, 18 EDS-HAT surveillance isolates collected between November 2016 and February 2018 were distributed along the entire subclade F branch (Fig. 1, red branches). The subclade F isolates were highly genetically related, with a median of 13 cgSNPs (range, 0 to 38) between isolate pairs. These data suggest the persistence and continued transmission of an emergent lineage (clade II, subclade F) in our hospital.

### Time-calibrated phylogeny.

**FIG 2:**
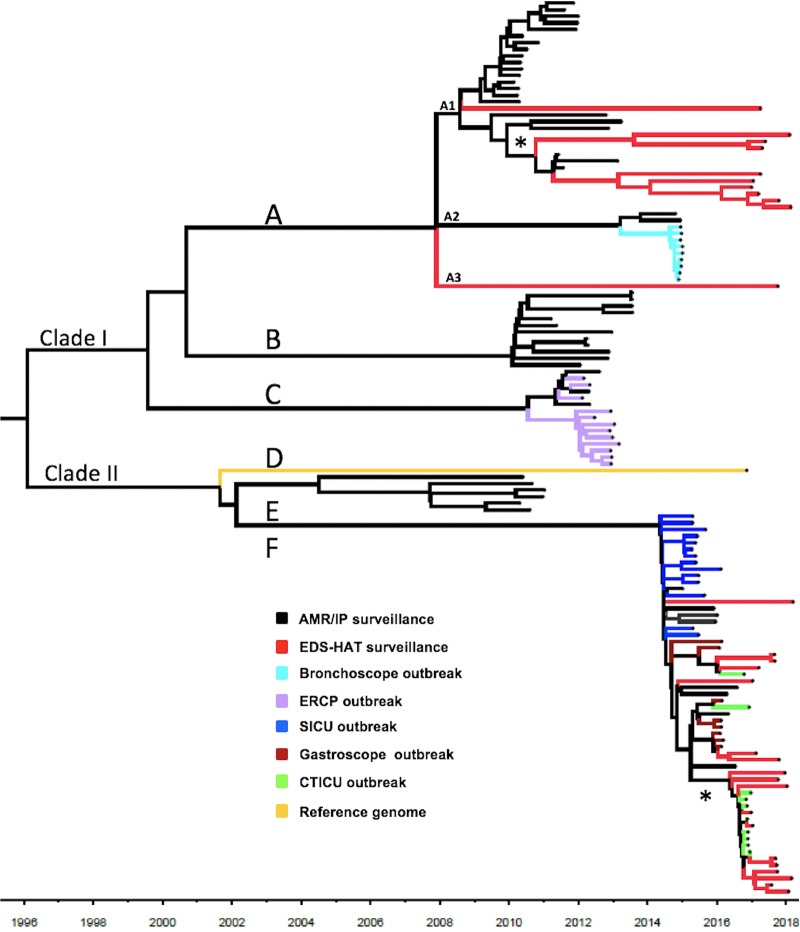
Time-calibrated phylogeny of 136 ST258 genomes. Outbreak-associated isolates are indicated with color coding as follows: turquoise, bronchoscope; lavender, ERCP; blue, SICU; brown, gastroscope; green, CTICU. Red branches represent EDS-HAT contemporary surveillance isolates. Black branches represent AMR/IP surveillance isolates. Letters indicate the subclades and sublineages, Asterisks (*) indicate the emerging A1 and F sublineages.

A regression of root-to-tip divergence against time demonstrated sufficient temporal signal within the data set to implement a time-calibrated phylogenetic analysis (17) (Fig. S1B). Using Bayesian analyses, a clock rate for the ST258 population in our study was calculated at 1.9 × 10^−6^ substitutions per site per year (95% highest posterior density, 1.7 × 10^−6^ to 2.1 × 10^−6^), which was consistent with previous studies (18, 19). The resulting time-calibrated phylogeny suggested that the ST258 population diverged into clades I and II around 1996 (Fig. 2), consistent with prior estimates (18). Subsequently, clades I and II each diverged into three subclades, as described above, with the most recent common ancestor(s) (MRCA) occurring between 2000 and 2002. Within clade I, subclade A was predicted to have emerged in our hospital in 2008 and a subset, A1*, has persisted (Fig. 2, branches A1 and A1*). Subsequently, subclades B and C emerged between 2010 and 2012 (Fig. 2, branches B and C). While neither subclade B nor subclade C persisted, isolates belonging to the A1 lineage continued to be observed throughout the 8-year study period. In contrast, outbreaks associated with clade II were not observed in our hospital prior to 2015. Clade II isolates belonging to subclade F were first observed in the SICU beginning in December of 2014 (Fig. 2, branch F). Subsequently, subclade F emerged as a dominant lineage associated with multiple outbreaks from 2015 through 2017 (Fig. 1; see also Fig. 2). In addition, subclade F comprised the majority (60.7%, 17/28) of the contemporary EDS-HAT surveillance isolates collected from 2016 to 2018 (Fig. 2, branch F, red data). To date, no epidemiologic evidence has been found linking these EDS-HAT CRKP infections to one another. Within subclade F, sublineage F* represents an emergent clone (Fig. 2, branch F*). Together, these data demonstrate the dynamic changes in the ST258 population structure over the 8-year study period and highlight the successive waves of distinct ST258 subpopulations in our hospital.

### Pan-genome analysis.

**FIG 3:**
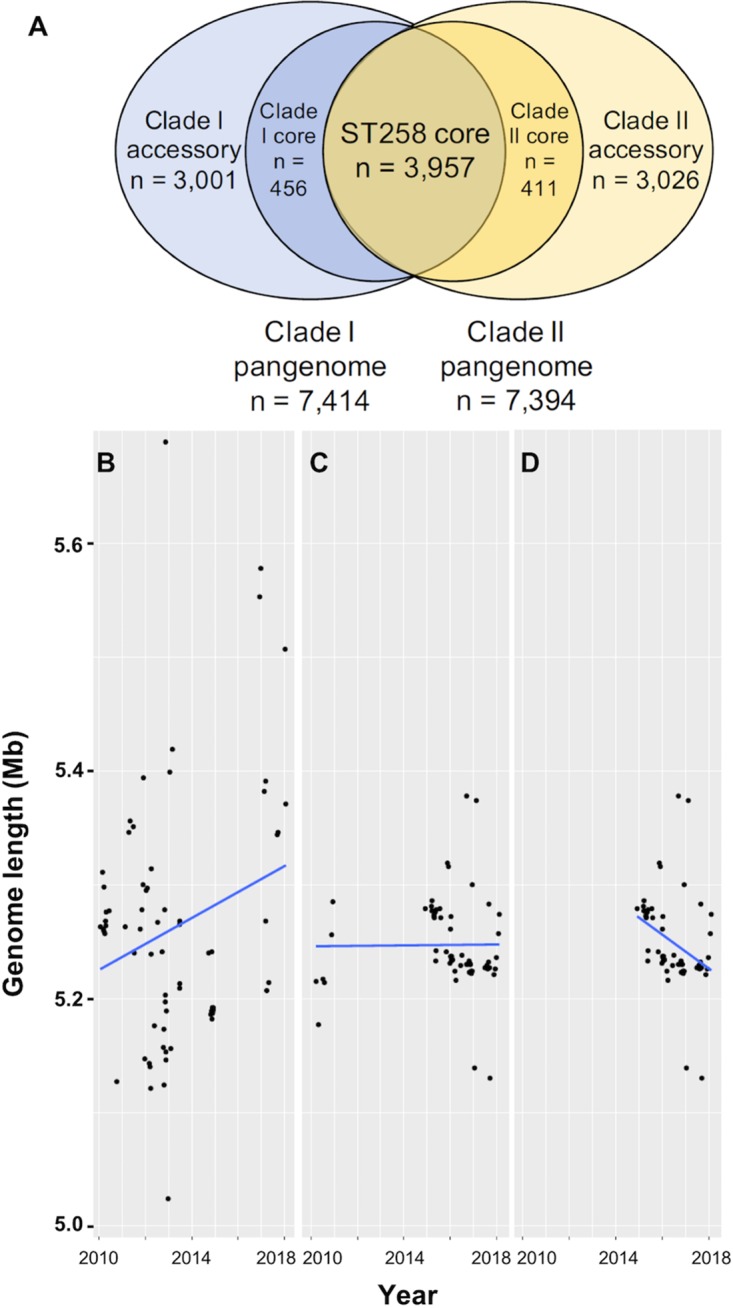
Pan-genome analysis. (A) Venn diagram illustrating numbers of shared core genes and unique core and accessory genes in clade I and clade II isolate genomes. (B to D) Linear regressions of genome size over time (years) in clade I (B), clade II (C), and subclade F (D). The genome size (in megabases) of each isolate is plotted versus the culture date.

The pan-genomes of the study isolates were investigated to identify the gene content differences that distinguished the clades from one another. The pan-genomes of clades I and II were similar in size (Fig. 3A); however, we identified a large number of genes that were enriched in one clade or the other (Table S3). In general, differences in gene content between clade I and clade II were largely associated with capsular gene loci and mobile genetic elements, including plasmids, integrative conjugative elements (ICEs), and prophages (Table S3), as has been previously described (18, 20). While the core and accessory genomes of each clade were similar in size, differences in genome size were observed between the two clades over time (Fig. 3). The genome size of clade I isolates increased significantly over time (linear regression *P = *0.03), while the change in genome size of clade II isolates as a whole was not statistically significant (*P = *0.24) (Fig. 3B and C). Subclade F genome sizes, however, tended to decrease over time (linear regression *P ≤ *0.01) (Fig. 3D). Together, these data suggest that clade I isolates have been diversifying over time, while clade II subclade F isolates might be specializing by reducing their genome size.

10.1128/mBio.01945-19.8 TABLE S3 Genes enriched between different subclades. Download Table S3, XLSX file, 0.1 MB. Copyright © 2019 Marsh et al. 2019 Marsh et al. This content is distributed under the terms of the Creative Commons Attribution 4.0 International license.

Because we saw temporal changes in genome size in both clade I and clade II isolates, we next identified genes that were present or absent in the persistent subclade A1 and F isolates. This analysis identified a 138-kb K. pneumoniae integrative conjugative element 10 (ICE*Kp10*), which was present in emergent subclade A1* isolates and in all but two subclade F isolates (Fig. S2; see also Table S3). The element is integrated into the chromosome in the closed KLP155 and KLP157 genome assemblies and harbors the genes necessary for synthesis of two virulence determinants, namely, the iron siderophore yersiniabactin and the genotoxin colibactin (21, 22). The presence of these virulence factors and the fact that ICE*Kp10* was found in emergent persistent subclades in our hospital suggest that this element provides ST258 CRKP with a selective advantage over isolates that lack the element.

10.1128/mBio.01945-19.2 FIG S2 RAxML phylogeny of 136 ST258 K. pneumoniae genomes with bootstrap values of >90 indicated. Subclades and sublineages are labeled alphanumerically. ICE*Kp10* presence indicated by the heat map is defined by percent coverage of ICE*Kp10* for hits with >95% identity by BLAST. Download FIG S2, PDF file, 0.1 MB. Copyright © 2019 Marsh et al. 2019 Marsh et al. This content is distributed under the terms of the Creative Commons Attribution 4.0 International license.

### Antimicrobial resistance genes.

**FIG 4:**
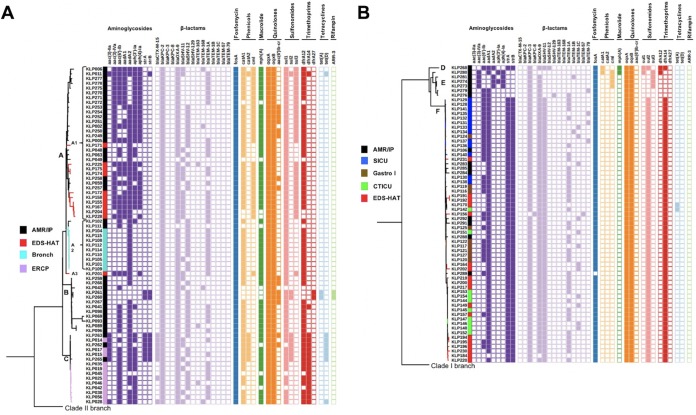
Presence/absence matrix of antimicrobial resistance genes among ST258 clade I (A) and clade II (B) isolates. Letters denote subclades A to F and sublineages (A1 to A3).

A BLAST search of all 136 genomes against the ResFinder database, the Comprehensive Antibiotic Resistance database (CARD), and the NCBI resistance gene database identified multiple AMR genes associated with resistance to aminoglycoside, β-lactam, chloramphenicol, fluoroquinolone, fosfomycin, macrolide, sulfonamide, tetracycline, and trimethoprim antibiotics (Fig. 4; see also Table S1). Clade I isolates showed greater AMR gene content and higher diversity in their AMR genes than clade II isolates. Clade I isolates had an average of 15.4 AMR genes, compared to 12.4 AMR genes observed among clade II isolates (*P <* 0.001) (Fig. 4; see also Table S1). The majority of clade I isolates carried the *aadA2* aminoglycoside resistance gene, while most clade II isolates harbored the *aadA1* allele. β-Lactamases belonging to the OXA, TEM, and SHV groups were present in both clade I and clade II ST258 isolates. The clade I isolates harbored *bla*TEM-1A predominantly, whereas the clade II isolates, despite being highly related to one another, were characterized by multiple distinct *bla*TEM alleles (Fig. 4). Clade I isolates harbored either *bla*SHV-11 or *bla*SHV-12 alleles, while clade II isolates were characterized by *bla*SHV-11. All clade I isolates carried the KPC-2 carbapenemase enzyme, while all clade II isolates carried the KPC-3 enzyme, except for one isolate that carried the KPC-8 enzyme instead. This KPC variant evolved in a patient following treatment with ceftazidime-avibactam (13). All isolates in both clades contained *oqxA* and *oqxB* genes, encoding resistance to quinolones, and all but one isolate (KLP289) contained the *fosA* fosfomycin resistance gene. Allelic differences in the genes encoding trimethoprim resistance between clades were also observed, with the majority of clade I isolates harboring the *dfrA12* (85.9%, 61/71) gene whereas clade II isolates carried predominantly *dfrA14* (90.8%, 59/65). Sulfonamide resistance among most clade II isolates was encoded by *sul2*, whereas the majority of clade I isolates carried *sul1*. Finally, chloramphenicol resistance genes *catA1* and *cml* were highly prevalent among clade I isolates, while the chloramphenicol resistance determinants were mostly absent from the clade II isolates and were entirely absent from the subclade F isolates (Fig. 4B).

Differences in the AMR gene content between different subclades and sublineages were also observed. Clade A1 isolates tended to harbor more aminoglycoside determinants than other clade I and clade II isolates. Most A1 isolates also harbored the *sul3* sulfonamide resistance gene. Interestingly, several recent EDS-HAT isolates that belong to the emergent A1 sublineage (KLP204 and KLP228) had fewer AMR genes, illustrating the dynamic nature and continued evolution of this clone (Fig. 4A). In clade II, the emergent subclade F isolates harbored multiple drug resistance determinants that were not present in subclade E isolates, which were all collected in 2010. For instance, subclade F genomes harbored *strAB*, *bla*SHV-11, *bla*TEM, *dfrA14* and *sul2* genes, which were all absent in clade E genomes (Fig. 4B). These data highlight the capacity of persistent subclade F strains to acquire and maintain multiple drug resistance elements over an extended period of time in hospitalized patients.

### KPC-encoding plasmids and additional plasmid replicons.

**FIG 5:**
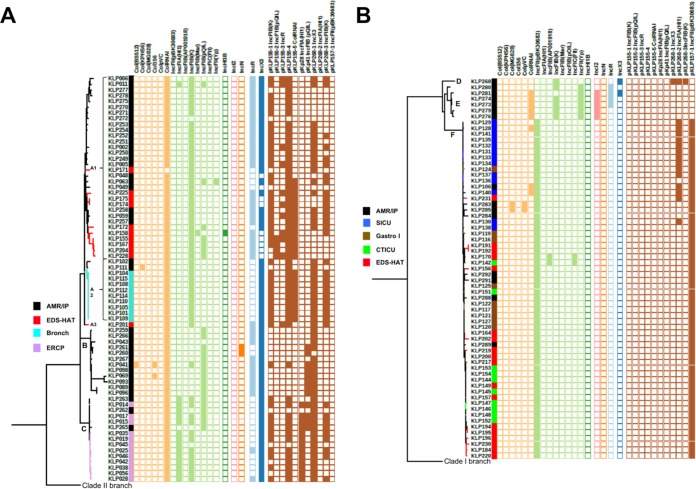
Presence/absence matrix of plasmid replicons present among ST258 clade I (A) and clade II (B) isolates. Additional letters denote subclades A to F and sublineages (A1 to A3). The presence of Col-type and Inc-type replicons is indicated at the left, and the presence of entire plasmids from selected isolates is indicated at the right.

Similarly to their AMR gene content, plasmid content also differed between and within the ST258 clades. Consistent with their subclade structure, ST258 clade I isolates demonstrated greater plasmid replicon content and diversity than clade II isolates. Clade I isolates carried an average of 5.0 plasmid replicons compared to the 1.9 plasmid replicons harbored among clade II isolates (*P < *0.001). BLAST analysis of the PlasmidFinder database revealed that most of the clade I isolates carried ColRNAI, IncFIB (K), IncFII, IncR, and IncX3 replicons (Fig. 5A). Long-read sequencing performed with Oxford Nanopore Technology and subsequent hybrid assembly of a representative clade I isolate (KLP155) identified five distinct plasmids belonging to five incompatibility groups, including IncFIB(K), IncFIB(pQIL), IncFII, IncR, and ColRNAI (Table 1). The 172-kb IncF1B(K) plasmid pKLP155-1 was found to encode KPC-2 on a Tn*4401a* element that showed >99.9% sequence identity to pBK32179 (23). Plasmid pKLP155-1 was present in most clade I isolates (Fig. 5A); isolates that lacked pKLP155-1 harbored IncFIB(pQIL) and/or IncFIA(HI1) replicons, both of which can encode KPC-2, as we demonstrated previously (2) (Fig. 5A). For instance, KPC-encoding plasmid pKp28 belonging to IncFIA(HI1) was present in the subclade C ERCP-associated outbreak isolates, but this plasmid was not identified in any other subclades (Fig. 5A). Similarly, IncFIB(pQIL) plasmid pKp41was observed only transiently in subclade B and C isolates. The majority of contemporary EDS-HAT isolates belonging to the emergent A1 group did not harbor the IncX3 replicon but contained an IncFIB(pQIL) replicon. These data illustrate the dynamic nature and complexity of clade I plasmids and the propensity of clade I isolates for maintaining multiple plasmid replicons.

**TABLE 1:** Characteristics of clade I and clade II plasmids in this study

Plasmid	Clade (subclade)	Size (bp)	Incompatibility group(s)	AMR genes
pKLP155-1	I (A1)	172,196	IncFIB(K)	*aadA2*, *aph(3')-Ia*, *blaKPC-2*, *blaTEM-1A*, *blaOXA-9*, *catA1*, *dfrA12*, *mphA*, *sul1*
pKLP155-2	I (A1)	96,242	IncFIB(pQIL)	None
pKLP155-3	I (A1)	45,730	IncR	*aac(3)-IVa*, *aac(6')-Ib*, *aadA1*, *aadA2*, *aph(4)-Ia*, *cml*, *sul3*
pKLP155-4	I (A1)	29,709	Not found	None
pKLP155-5	I (A1)	9,506	ColRNAI	Colicin immunity protein-encoding gene
pKp41	I (B)	113,626	IncFIB(pQIL)	*blaKPC-2*, *blaTEM-1A*, *blaOXA-9*
pKp28	I (C)	83,399	IncFIA(HI1)	*blaKPC-2*, *blaTEM-1A*, *blaOXA-9*, *dfrA14*
pKLP268-1	II (D)	43,370	IncX3	*blaSHV-12*
pKLP268-2	II (D)	71,802	IncFIA(HI1)	*aac(6')-Ib*, *aadA1*, *blaKPC-3*, *blaOXA-9*, *dfrA14*, *strAB*, *sul2*
pKLP268-3	II (D)	212,419	IncFIB(K)	*aadA2*, *catA1*, *dfrA12*, *mphA*, *sul1*
pKLP157-1	II (F)	172,181	IncFII(pBK30683)	*aac(6')-Ib*, *aadA1*, *blaKPC-3*, *blaSHV-11*, *blaTEM-1A*, *blaOXA-9*, *dfrA14*, *strAB*, *sul2*

In comparison, the plasmid replicon content of clade II isolates was less diverse than that of clade I isolates. The subclade D KLP268 reference isolate had three replicons [IncFIA(HI1), IncFIB(K), and IncX3] (Fig. 5B; see also Table 1). Plasmid pKLP268-2, a 71-kb IncFIA(HI1) plasmid, encoded KPC-3 on a Tn*4401a* element that showed 99.75% sequence identity to pBK32533 (24). This plasmid was also present in a subset of subclade F isolates belonging to the SICU outbreak but was not present in gastroscopy, CTICU, or contemporary EDS-HAT isolates (Fig. 5B). None of the KLP268 reference plasmids were found in subclade E isolates, but plasmid replicon diversity was evident among these isolates. Six different replicons were observed among subclade E isolates, with some isolates having as many as five replicons present (Fig. 5B). All but one subclade E isolate harbored an IncI2 replicon, which has been shown to encode KPC-3 (25). In addition, subclade E isolates were unique in that they all harbored IncFII(Yp) replicons. The majority of subclade F isolates harbored a single IncFII(pBK30683) replicon (Fig. 5B). This replicon likely corresponds to the KPC-3-encoding, 172-kb IncFII(pBK30683) plasmid contig pKLP157-1, which was identified from long-read sequencing and hybrid assembly of a representative clade II isolate, KLP157. While a few subclade F isolates harbored additional plasmid replicons (Fig. 5B), overall, their replicon profiles were consistent with the clonal subclade F population structure and further support our theory that subclade F genomes are undergoing specialization and pan-genome reduction.

### Genomic epidemiology of subclade F.

**FIG 6:**
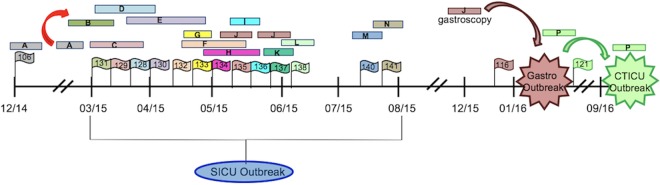
Timeline of subclade F transmission. Colored bars represent patient stays, and colored flags indicate CRKP isolates collected from the corresponding patient infections. Flag labels correspond to the KLP isolate number. The red arrow denotes transmission from patient A (via a shared hospital unit location) to patient B on the SICU, the brown arrow denotes patient J as the index case for the gastroscopy outbreak, and the green arrow denotes patient P as the index case for the CTICU outbreak.

Subclade F was characterized by three outbreaks that occurred over a 3-year period. The first observed clade II, subclade F isolate was collected from patient A in December of 2014 (KLP106) (Fig. 6). A K. pneumoniae SICU outbreak began in March of 2015 and was subsequently traced from patient A to patient B through a shared hospital unit location (Fig. 6, red arrow) (16). Ultimately, subclade F strains were transmitted through the SICU to patient J, who underwent gastroscopy in December of 2015 (Fig. 6, brown bar). CRKP isolates obtained from patient J during the SICU stay (KLP135) and after gastroscopy (KLP116) were highly genetically related to patient isolates from the gastroscopy outbreak, with <10 cgSNPs between isolate pairs (Table S1; see also Table S2). These genomic epidemiology data traced the gastroscopy outbreak to the introduction of subclade F K. pneumoniae from patient A in 2014. The gastroscopy outbreak was linked to the CTICU cluster through patient P, who developed a CRKP infection following gastroscopy and was transferred to the CTICU in September 2016. The clinical isolate from patient P (KLP121) was highly genetically related to subsequent isolates obtained from patients residing on the CTICU who shared common rooms and staff members (Fig. 6) (Table S1; see also Table S2). Ongoing EDS-HAT surveillance has continued to identify isolates that are highly genetically related to subclade F but without additional epidemiologic links. The propensity of subclade F for persistence over a 4-year period suggests that this ST258 population may be better able to survive and/or be more readily transmitted within the hospital and among hospitalized patients than other ST258 subclades.

10.1128/mBio.01945-19.7 TABLE S2 Numbers of cgSNPs between isolate pairs. Download Table S2, XLSX file, 0.1 MB. Copyright © 2019 Marsh et al. 2019 Marsh et al. This content is distributed under the terms of the Creative Commons Attribution 4.0 International license.

### Genotypes associated with persistent ST258 sublineages.

An investigation of cgSNP differences across the time-calibrated phylogeny was performed to identify genetic mutations potentially associated with the emergence and persistence of clade I and clade II sublineages. Isolates on a well-supported sublineage A1 branch that contained 9 of the 11 most contemporary isolates shared missense mutations in four genes that distinguished them from other subclade A isolates; the mutated genes were associated with DNA methylation, membrane transport, and nitrogen metabolism (Table 2) (Fig. 2, branch A1*). Consistent with the long branch separating subclade F from subclades D and E, all F isolates harbored 61 cgSNPs that distinguished them from other clade II isolates (Table S4). Many (33/61, 54%) of these mutations were nonsynonymous and resided in genes encoding proteins that likely function in biofilm formation, iron transport, membrane efflux, attachment, and transcriptional regulation (Table S4). Similarly to sublineage A1, a well-supported subclade F branch that contained 11 of the 18 most contemporary isolates harbored unique mutations in five genes that distinguished them from other subclade F isolates (Table 2) (Fig. 2, branch F*). Several of these genes encoded proteins necessary for adaptive responses to different environments, including YfgF, a predicted regulator of the cyclic-di-GMP signaling pathway, and FecA, an iron transport protein (Table 2). Together, these mutations may provide survival advantages to ST258 sublineages during infections or in the hospital environment.

**TABLE 2:** Genes with cgSNPs unique to persistent subclade A1 and F isolates

Gene	Predicted function	Amino acid substitution	Branch
*dcm*	DNA-cytosine methyltransferase	A111T	A1*
*tauB*	Taurine import ATP-binding protein	V75A	A1*
*ybaL*	Inner membrane protein	A83T	A1*
*KLP00268_05120*	Nitronate monooxygenase	indelYYR^*a*^	A1*
*yfgF*	Cyclic di-GMP phosphodiesterase	I211V	F*
*serA*	d-3-Phosphoglycerate dehydrogenase	A360Q	F*
*mtnC*	Enolase phosphatase E1	A57S	F*
*alkH*	Aldehyde dehydrogenase	T227N	F*
*fecA*	Fe^+3^ dicitrate transport	Q293^*a*^	F*

a Stop codon.

10.1128/mBio.01945-19.9 TABLE S4 Characteristics of cgSNPs unique to subclade F isolates. Download Table S4, XLSX file, 0.01 MB. Copyright © 2019 Marsh et al. 2019 Marsh et al. This content is distributed under the terms of the Creative Commons Attribution 4.0 International license.

### Phenotypes associated with persistent ST258 sublineages.

To assess phenotypes that might contribute to persistence and virulence of different ST258 lineages, all study isolates were tested for biofilm production and mucoviscosity. While modest levels of biofilm production were observed in most isolates, robust biofilm formation was observed among the six isolates belonging to clade II, subclade E (Fig. S4A). One isolate (KLP281) formed a particularly dense biofilm at the bottom of the assay plate; examination of cgSNPs unique to this isolate identified a premature stop codon in the bifunctional ppGpp synthase/hydrolase SpoT enzyme, which is known to play a role in biofilm formation (26). Mutations in *spoT* that affect ppGpp can be drivers of AMR in a biofilm-dependent manner (27). These data suggest that increased biofilm formation was not a driving force behind the evolution of ST258 persistence in our hospital but might affect individual isolates. Similarly, we did not observe large differences in mucoviscosity among ST258 isolates, with the exception of one subclade A strain (KLP278) that was noticeably more mucoviscous than other strains (Fig. S4B). SNPs unique to this strain included nonsynonymous cgSNPs in a *ptrA* protease, a hypothetical protein, and a *gltC* transcriptional regulator. Overall, despite observations of variants predicted to impact attachment and biofilm production, particularly in the subclade F genomes, neither biofilm formation levels nor mucoviscosity levels (as measured *in vitro*) were increased among the persistent ST258 populations within our hospital.

10.1128/mBio.01945-19.4 FIG S4 (A) Biofilm formation among 136 K. pneumoniae ST258 isolates. Assay data are plotted against the core genome phylogeny, with the bars indicating the mean OD_595_ ± standard error of results from three biological replicates, each including up to eight technical replicates. (B) Mucoviscosity among 136 K. pneumoniae ST258 isolates. Assay data are plotted against the core genome phylogeny, with mucoviscosity indicated by bars representing the mean postspin/prespin OD_595_ ratios ± standard errors of results from at least three biological replicates. Download FIG S4, PDF file, 0.2 MB. Copyright © 2019 Marsh et al. 2019 Marsh et al. This content is distributed under the terms of the Creative Commons Attribution 4.0 International license.

**FIG 7:**
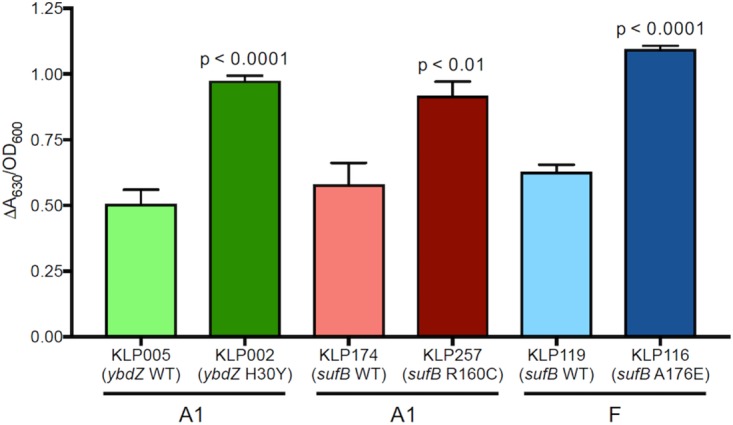
Iron binding of genetically similar pairs of sublineage A1 and subclade F isolates that differ in *ybdZ* and *sufB* alleles. The ratio of absolute change in *A*_630_ to bacterial culture OD_600_ is plotted, with higher ratios corresponding to higher levels of iron binding. *P* values represent the results of unpaired *t* tests comparing each mutant strain with the corresponding strain without the mutation (i.e., the wild type [WT]).

Mutations in iron binding and transport genes were also observed among clade I and clade II isolates. These included nonsynonymous SNPs and nonsense mutations in the iron-sulfur cluster assembly genes *sufB* and *sufD*, the ferrichrome-iron receptor *fhuA* gene, the catecholate siderophore receptor *fiu* gene, the ferric aerobactin receptor *iutA* gene, the ferric enterobactin receptor *pfeA* gene, and the enterobactin biosynthesis protein-encoding *ybdZ* gene (Table S5). Pairs of genetically similar isolates with allelic differences at these loci were selected to test for phenotypic differences in iron binding. Isolates with mutations in *ybdZ* and *sufB* showed increased iron binding compared to their wild-type counterparts (Fig. 7). Of note, the mutations in *sufB* that correlated with increased iron binding were present among isolates belonging to persistent populations in both A1 and F. Moreover, the *sufB* mutations found in these strains differed between sublineage A1 and subclade F, suggesting that they arose independently in clade I and clade II isolates, perhaps because they contributed to the persistence of these distinct ST258 sublineages.

10.1128/mBio.01945-19.10 TABLE S5 Mutations in iron binding and transport genes. Download Table S5, XLSX file, 0.02 MB. Copyright © 2019 Marsh et al. 2019 Marsh et al. This content is distributed under the terms of the Creative Commons Attribution 4.0 International license.

## DISCUSSION

CRKP infections continue to be problematic in the health care setting. In particular, ST258 CRKP is of public health concern because of its widespread distribution, its multidrug resistance, and its ability to rapidly exchange plasmidborne resistance determinants with other *Enterobacteriaceae*. In this study, we examined the genomic epidemiology of ST258 isolates from a single tertiary care hospital collected over an 8-year period and identified both outbreak-associated and persistent lineages that appear to exhibit endemicity in this setting. Our data highlight the dynamic nature of ST258 at our institution, with both clade I and clade II isolates having caused repeated ward-associated and device-associated outbreaks over the last decade. Furthermore, while the clade I and clade II genomes appear to have emerged and adapted differently, strains of both clades continue to persist in our setting despite ongoing interventions.

The two-clade CRKP ST258 population structure evident in our data set is consistent with previous studies (10, 28). Despite their relatively recent divergence from one another, our findings suggest that the clade I and clade II populations have adapted in different ways. We identified differences in the accessory genomes of the two clades, with clade I isolates carrying more antimicrobial resistance genes and plasmids than clade II isolates. Clade I genomes also appear to be increasing in size over time, while clade II subclade F genomes, which include all contemporary isolates in clade II, are getting smaller over time. These findings are unique to our study and may explain why clade I and clade II strains continue to coexist in our hospital.

Adaptive mutations likely contribute to ST258 persistence among hospitalized patients. In a recent study investigating CRKP ST258 colonization in a single patient, multiple adaptive mutations were identified over a 4.5-year period that resulted in increased virulence and drug resistance (29). Several of the mutated genes identified in that study, including *pfeA* and *rcsA*, were also mutated in subclade F isolates. These genes encode proteins involved in iron uptake and stress response signaling, respectively, and provide functions that are important for bacterial survival (30). We also identified additional subclade F mutations in genes that function in fimbrial biogenesis, biofilm formation, and iron transport. While increased *in vitro* biofilm formation was not observed in persistent subclades, we did observe increased levels of iron binding that correlated with mutations in the *sufB* and *ybdZ* genes. The SufB protein is required for assembly of the sulfur utilization factor (SUF), an iron-sulfur cluster assembly system that is critical for bacterial survival under iron-limiting conditions and is well conserved across the tree of life (31). Similarly, *ybdZ* is involved in the synthesis of the enterobactin siderophore produced by ST258 strains (32). While the precise role of these iron utilization proteins in K. pneumoniae infections has not been established, these data suggest that iron acquisition may be important for ST258 survival and persistence.

The epidemiology of ST258 at our hospital supports the idea of a pattern of clonal dominance and extinction over time. Several clade I endoscope-associated outbreaks (A2 and C isolates) persisted until the endoscopes were removed from service. Since then, neither subclade has been observed in our hospital. Clade I subclade B disappeared at approximately the same time as the ERCP intervention (endoscope removal), and this subclade has not been observed again. In contrast, subclade A1 isolates were observed among patients in our hospital throughout the study period from 2010 to 2018, and this population appears to be evolving with the emergence of A1* isolates. The recently emerged clade II F subclade has caused multiple outbreaks since 2014, and ongoing surveillance shows that it continues to be observed in hospitalized patients. While subclades A1* and F may represent continued reintroductions from the community to our institution, further investigations to identify previously unrecognized sources and transmission routes are required and are a major focus of our ongoing EDS-HAT study (33–35).

Our epidemiology findings demonstrate ongoing transmission and persistence of multiple ST258 lineages over time. Of concern is the identification of ICE*Kp10* harboring yersiniabactin and colibactin synthesis operons among emerging sublineages in our hospital. Yersiniabactin is an iron siderophore that enhances bacterial survival in the host (36). Colibactin is a genotoxin that induces DNA cross-linking in eukaryotic cells and disrupts host immune response (37). Both virulence factors are associated with K. pneumoniae lineages that cause invasive disease such as pyogenic liver abscess (38, 39). Recently, the convergence of drug resistance and virulence determinants among important CRKP lineages was demonstrated through a plasmid acquisition event in a fatal ST11 outbreak in China in 2016 (40). Furthermore, a study describing the genetic diversity of ICE*Kp* in a global collection of ST258 genomes has suggested a similar threat, whereby yersiniabactin and colibactin coalesce on the chromosome to generate hypervirulent CRKP lineages that pose a greater risk to patient populations (21). Thus, the emergence of CRKP ST258 bearing ICE*Kp10* is of concern and highlights the need for enhanced genomic surveillance of these serious infections.

There were several limitations of this study. For instance, isolate selection and inclusion criteria were not systematic. The retrospective sampling of available clinical isolates brought together isolates collected for a variety of different studies at our institution. Thus, the data presented may be affected by sampling biases that have obscured epidemiologic and evolutionary signals. Prospective sampling of hospital-acquired infections beginning in November of 2016, however, has been consistent and has allowed us to observe temporal succession and differing levels of persistence of discrete ST258 subclades over the course of the study. By combining retrospective and systematic prospective isolate collections, this study provided new insights into the dynamic changes occurring among ST258 populations in a single hospital over time. We are now validating the combination of machine learning of electronic health records and WGS surveillance in our EDS-HAT project in an attempt to identify and interrupt transmission of CRKP ST258 and other pathogens associated with serious disease in our hospital (33–35). Tracking of multidrug-resistant and emergent hypervirulent bacterial lineages through WGS surveillance is critical to infection prevention. Early detection permits rapid implementation of interventions to prevent further transmission, which in turn reduces patient morbidity and mortality as well as health care costs (35, 41).

### Conclusions.

The genomic epidemiology of ST258 at our institution over an 8-year period illustrates the emergence and dominance of distinct CRKP clones, which have evolved over time in different ways. Some clones caused outbreaks that were subsequently eliminated, while other clones have persisted to the present day. These data highlight the evolution of endemic ST258 subpopulations among hospitalized patients and the continued challenge of preventing serious CRKP infections in the health care setting.

## MATERIALS AND METHODS

### Study isolates.

Isolates were selected from previously published collections of ST258 genomes (2, 10, 12, 13, 14, 15) and from our prospective genomic epidemiology surveillance project, EDS-HAT. A total of 136 ST258 K. pneumoniae isolates from patient clinical samples, surveillance rectal swabs, and endoscopes were collected between January 2010 and February 2018 at the University of Pittsburgh Medical Center Presbyterian Hospital (UPMC) (see Table S1 in the supplemental material). The collection included serial isolates from 18 patients, including pairs of isolates from 13 patients and 22 isolates from 5 patients (Table S1). Isolates were collected for routine IP surveillance (*n* = 17), for outbreak investigations (*n* = 53), for studies investigating the evolution of AMR (*n* = 37) (Table S1) (2, 10, 12, 13), and for the EDS-HAT project (*n* = 29) that began in November 2016. For EDS-HAT, clinical CRKP isolates were collected >72 h after admission. Carbapenem susceptibility was determined by Microscan and Kirby-Bauer disk diffusion using breakpoints as defined by the clinical microbiology laboratory. This study was approved by the University of Pittsburgh Internal Review Board and was classified as being exempt from human consent.

### Whole-genome sequencing.

All sequencing reads from previously published studies were generated using Illumina-based sequencing strategies and 50-bp, 75-bp, or 150-bp paired-end reads on MiSeq, NextSeq, HiSeq instruments (2, 10, 12, 13, 15). Resulting reads were downloaded from the Sequence Read Archive (SRA) and quality processed through our bioinformatics pipeline (described below). Seven genomes reported in a previous study by Shields et al. (13) had previously been quality trimmed using sickle (GitHub) and were *de novo* assembled with SPAdes; the resulting contigs were obtained from GenBank and used in subsequent genomic comparisons in our pipeline. Genomic DNA from unpublished isolates (*n* = 70) were sequenced on either an Illumina MiSeq or a NextSeq 500 sequencer using a modification of an Illumina Nextera library preparation kit (Illumina, San Diego, CA) (42). Reads were quality filtered and assembled *de novo* using SPAdes v3.11 (43). To be included in the study, genome assemblies had to have (i) average read depth of >40×, (ii) a genome length within 10% of that of the reference genome (5.3 Mb to 6.5 Mb), (iii) a total number of contigs below 300, and (iv) an *N*_50_ value of greater than 50 kb (Table S1). All genome assemblies were subjected to Kraken (v1) taxonomic sequence classification for species identification and to rule out contamination (44). Genomes were annotated using Prokka annotation software (45). The KLP268 reference isolate was subjected to single-molecule real-time sequencing on a PacBio RS II instrument at the Yale Center for Genome Analysis. The genome was assembled using the hierarchical genome assembly process (HGAP 3.0) and refined using the resequencing protocol in SMRT analysis v2.3 software. Clade-specific reference isolates KLP155 (clade I) and KLP157 (clade II) were sequenced on a MinION device (Oxford Nanopore Technologies, Oxford, United Kingdom). Nanopore reads were combined with Illumina reads and assembled using the Unicycler hybrid assembly program (46). Unless otherwise noted, all genomic analyses were conducted using default parameters.

### Phylogenetic analyses.

Queries of the Kraken (http://ccb.jhu.edu/software/kraken/) and *Klebsiella* multilocus sequence typing (http://bigsdb.pasteur.fr/klebsiella/) databases were performed to identify species and sequence type (ST), respectively, using data from the assembled contigs (44). Capsular biosynthesis gene and *wzi* alleles were determined by querying the pubmlst/kpneumoniae database (http://bigsdb.pasteur.fr/klebsiella/). Assemblies were aligned to the KLP268 PacBio reference genome, and cgSNPs were called using Snippy v3.1 (https://github.com/tseemann/snippy). Recombination was removed with ClonalFramML v1.11. A maximum likelihood phylogenetic tree was generated with RAxML v8.2.11 using a general time-reversible model with a categorical model of rate heterogeneity (GTR-CAT), Lewis correction of ascertainment bias, and 100 bootstrap replicates (47). The resulting phylogenetic tree (see Fig. S2 in the supplemental material) was used to estimate the most recent common ancestor (MRCA) using BEAST v1.10.4 (48). BEAST was run using a relaxed exponential clock model with a GTR model for nucleotide substitution and a coalescent constant population prior with 20 million states, sampling every 5,000 states, and a burn-in rate of 10%. The time-calibrated phylogeny with the 95% highest posterior densities supporting the topology of the tree is shown in Fig. S3. A BLAST search of ResFinder (49), the Comprehensive Antibiotic Resistance database (CARD) (50), NCBI, and PlasmidFinder (51) was performed on assembled genomes to identify genes encoding antimicrobial resistance and plasmid replicons. An 80% similarity cutoff value was used to define antimicrobial resistance gene presence and was calculated by multiplying percent sequence identity and percent coverage of the gene. Similarly, a 95% cutoff value was used for plasmid replicon presence. Core and accessory genomes were defined using Roary v3.8.2 with a 95% sequence identity cutoff value. Support for the A1* and F* branches was obtained using data from cgSNP alignments with recombination removed by the use of ClonalFrameML v1.11 with KLP155 and KLP157 complete genomes as the references, respectively. Maximum likelihood phylogenetic trees were generated using a general time-reversible model with GTR-CAT and 1,000 bootstraps (Fig. S5A and B, respectively). For gene presence/absence analyses, a “study” group and a “comparison” group of isolates were defined. A gene was defined as present if found in >80% of the study isolates and in <20% of the isolates in the comparison group. Similarly, a gene was defined as absent (depleted) if found in >80% of comparison group isolates and <20% of study isolates. GenBank accession number KY454634 was used for ICE*Kp10* comparisons and annotations.

10.1128/mBio.01945-19.3 FIG S3 Time-calibrated phylogeny with 95% highest posterior density. Download FIG S3, PDF file, 0.07 MB. Copyright © 2019 Marsh et al. 2019 Marsh et al. This content is distributed under the terms of the Creative Commons Attribution 4.0 International license.

10.1128/mBio.01945-19.5 FIG S5 (A) RAxML phylogeny of clade I, subclade A, using the KLP155 genome as reference. Bootstrap values of greater than 90 are shown. Branches are color coded by epidemiology results as follows: black, AMR/IP surveillance isolates; red, EDS-HAT contemporary surveillance isolates; turquoise, bronchoscope-associated outbreak. The scale bar represents the number of substitutions per site. (B) RAxML phylogeny of clade II, subclade F, using the KLP157 genome as reference. Bootstrap values greater than 90 are shown. Branches are color coded by epidemiology results as follows: black, AMR/IP surveillance isolates; blue, SICU outbreak isolates; brown, gastroscope-associated outbreak isolates; green, CTICU-associated outbreak isolates; red, EDS-HAT contemporary surveillance isolates. The scale bar represents the number of substitutions per site. Download FIG S5, PDF file, 0.2 MB. Copyright © 2019 Marsh et al. 2019 Marsh et al. This content is distributed under the terms of the Creative Commons Attribution 4.0 International license.

### Phenotypic assays.

Biofilm formation was measured using a standard assay with minor modifications (52). Briefly, bacteria were grown overnight in brain heart infusion (BHI) media containing 0.25% glucose in 96-well polyvinyl chloride plates. The next day, adherent cells were washed and incubated in 0.1% crystal violet solution followed by incubation with 4:1 ethanol/acetone solution, and optical density at 595 nm (OD_595_) was read. Higher absorbance readings correspond to higher levels of biofilm formation. Biofilm assays were conducted on at least three biological replicates performed with up to eight technical replicates each. Negative controls containing media only were included in each biological replicate.

Mucoviscosity was quantified by growing each isolate overnight in LB media at 37°C without agitation. Each culture was resuspended to homogeneity, and the OD_595_ was measured and recorded as the “prespin” OD. Cultures were centrifuged, supernatant was removed, and OD_595_ was measured and recorded as the “postspin” OD. The ratio of postspin OD to prespin OD was calculated for each strain, with higher ratios corresponding to greater mucoviscosity. Data were averaged over at least three biological replicates.

Levels of iron binding was measured using the chrome azurol S assay (53). Briefly, bacterial strains were incubated in M9 minimal media for 24 h at 37°C. The OD_600_ of each culture was recorded, filtered culture supernatants were mixed with freshly prepared chrome azurol S (CAS) reagent, and the OD_630_ was measured for each bacterial sample and for the medium-only control. Iron binding was quantified by calculating the following ratio: *A*_630_ (control sample)/OD_600_.

### Statistical methods.

Trends in genome size over time were assessed with linear regression using Stata v15.1. Two-tailed *t* tests were used to assess differences in iron binding.

### Data availability.

The raw sequence reads and selected assemblies listed in Table S1 are available from the Sequence Read Archive and assembly database at the National Center for Biotechnology Information. Reads for 40 previously sequenced isolates were downloaded from the NCBI Sequence Read Archive (SRA) (the accession numbers are listed in Table S1). The 96 newly sequenced ST258 genomes have been deposited in the SRA under the accession numbers listed in Table S1. Completed and annotated reference genome assemblies for KLP155 and KLP157 have been deposited into BioProject PRJNA475751 as BioSamples SAMN10435697 and SAMN10435699, respectively. The reference genome assembly for KLP268 has been deposited as BioSample SAMN12588286 in BioProject PRJNA529592.
