# Supplementary figures and images for: Evolution of Outbreak-Causing Carbapenem-Resistant Klebsiella pneumoniae ST258 at a Tertiary Care Hospital over 8 Years
Source: mBio. 2019 Sep 3;10(5):e01945-19. doi: 10.1128/mBio.01945-19 (PMC6722418; doi:10.1128/mBio.01945-19)

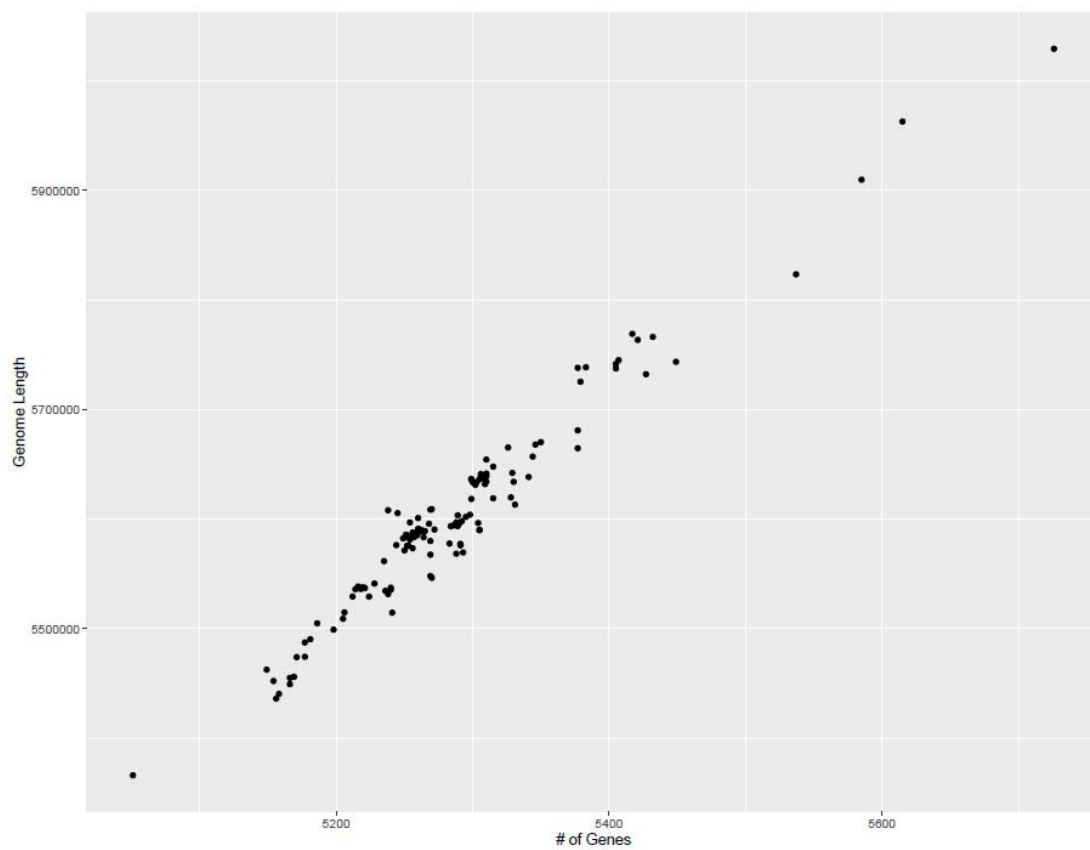

**Fig. S1A.**

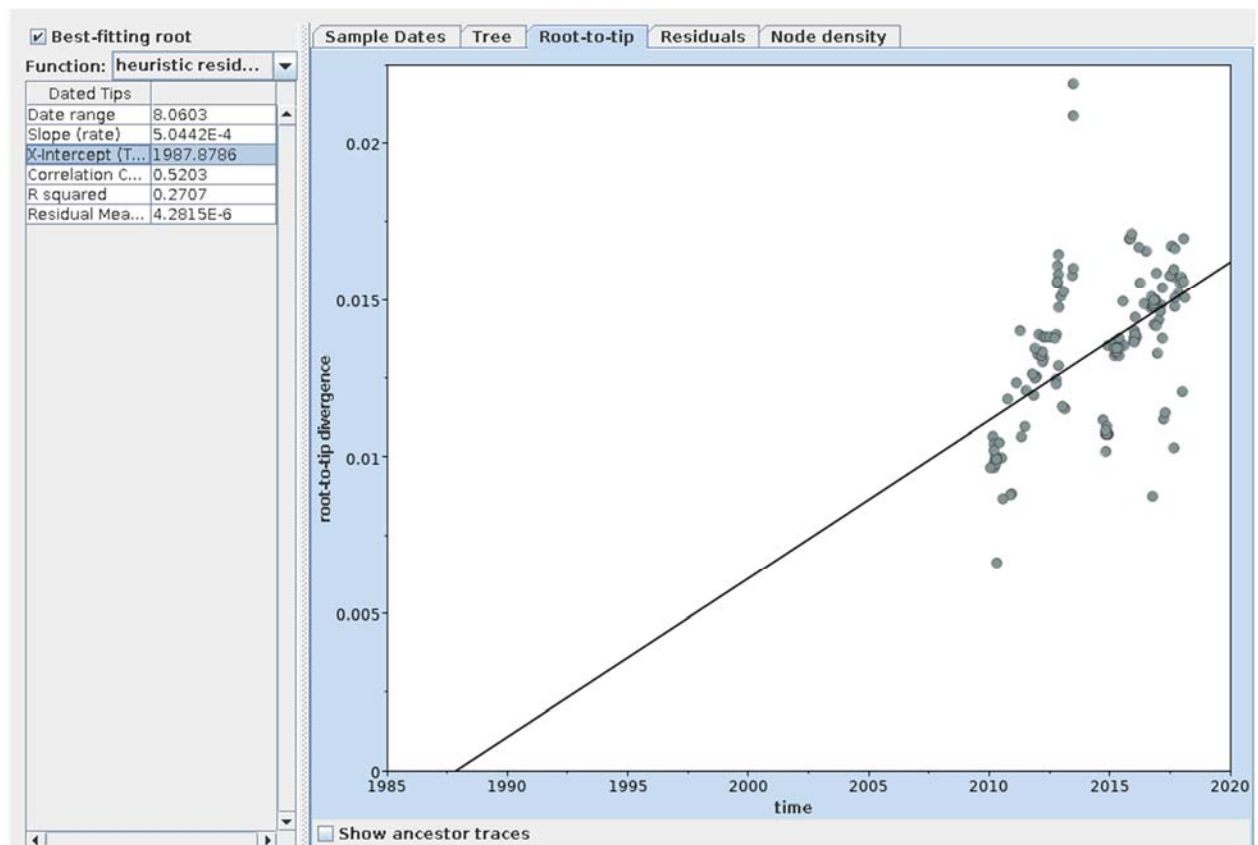

**Fig. S1B.**

Supplement: FIG S1 [file mBio.01945-19-sf001.pdf]

Tree scale: 0.001

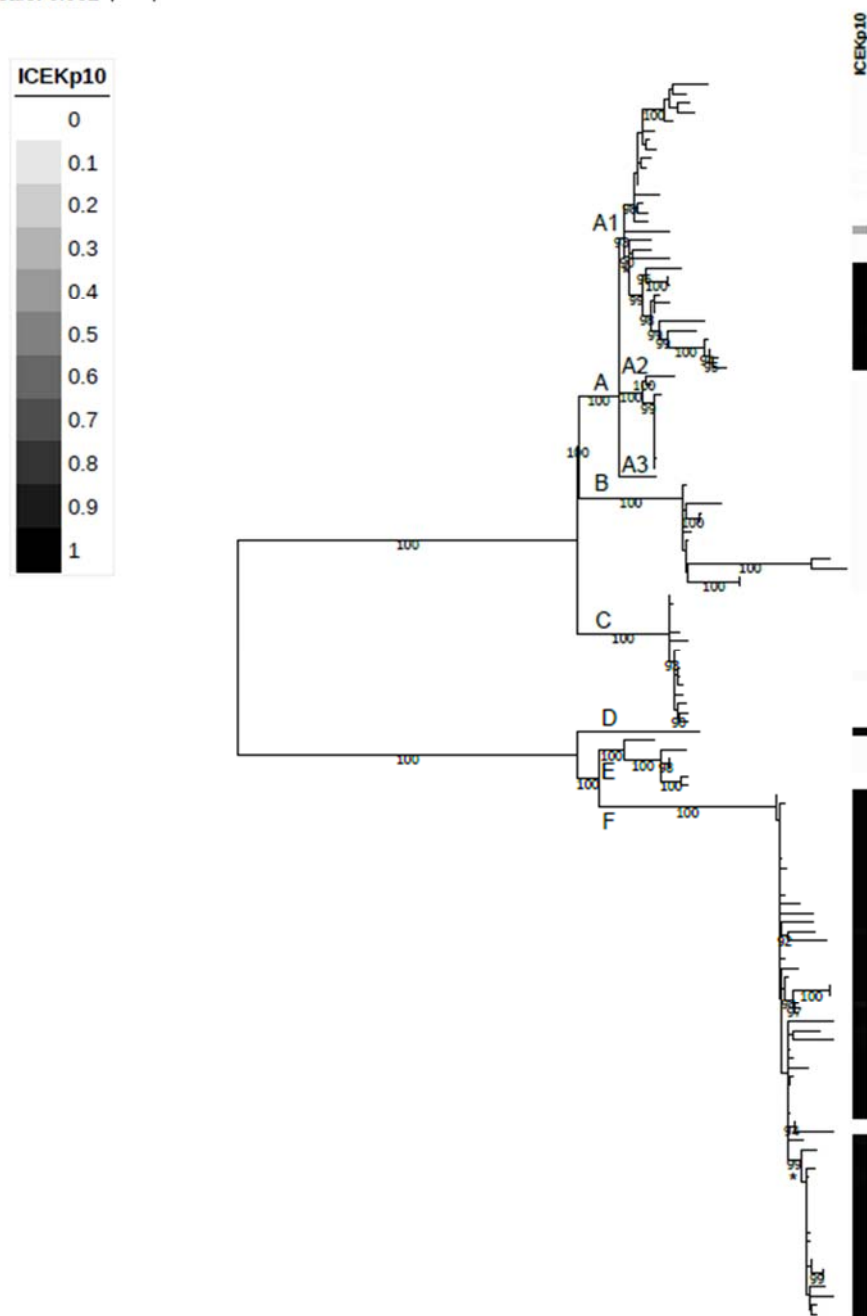

Fig. S2

Supplement: FIG S2 [file mBio.01945-19-sf002.pdf]

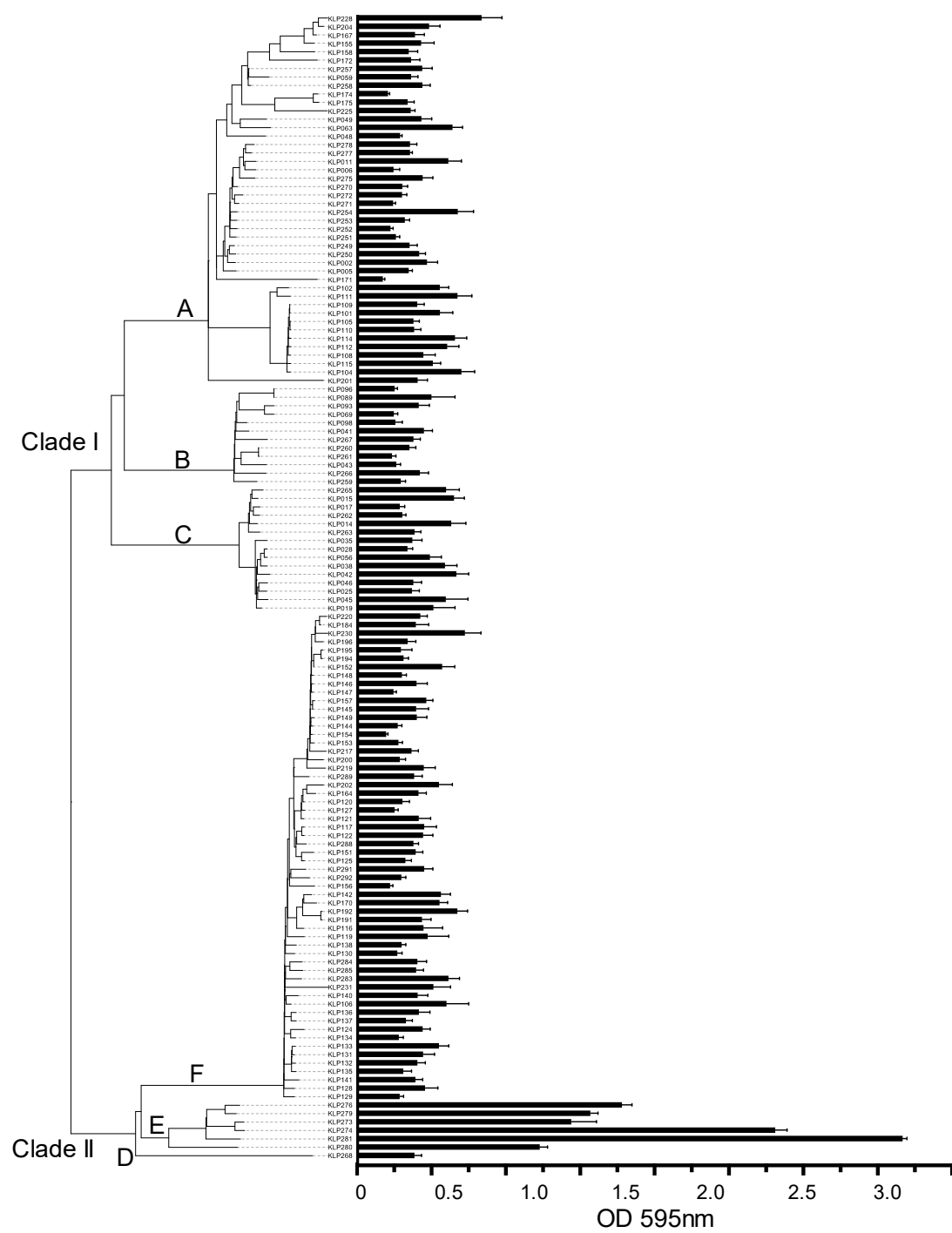

**Fig. S4A**

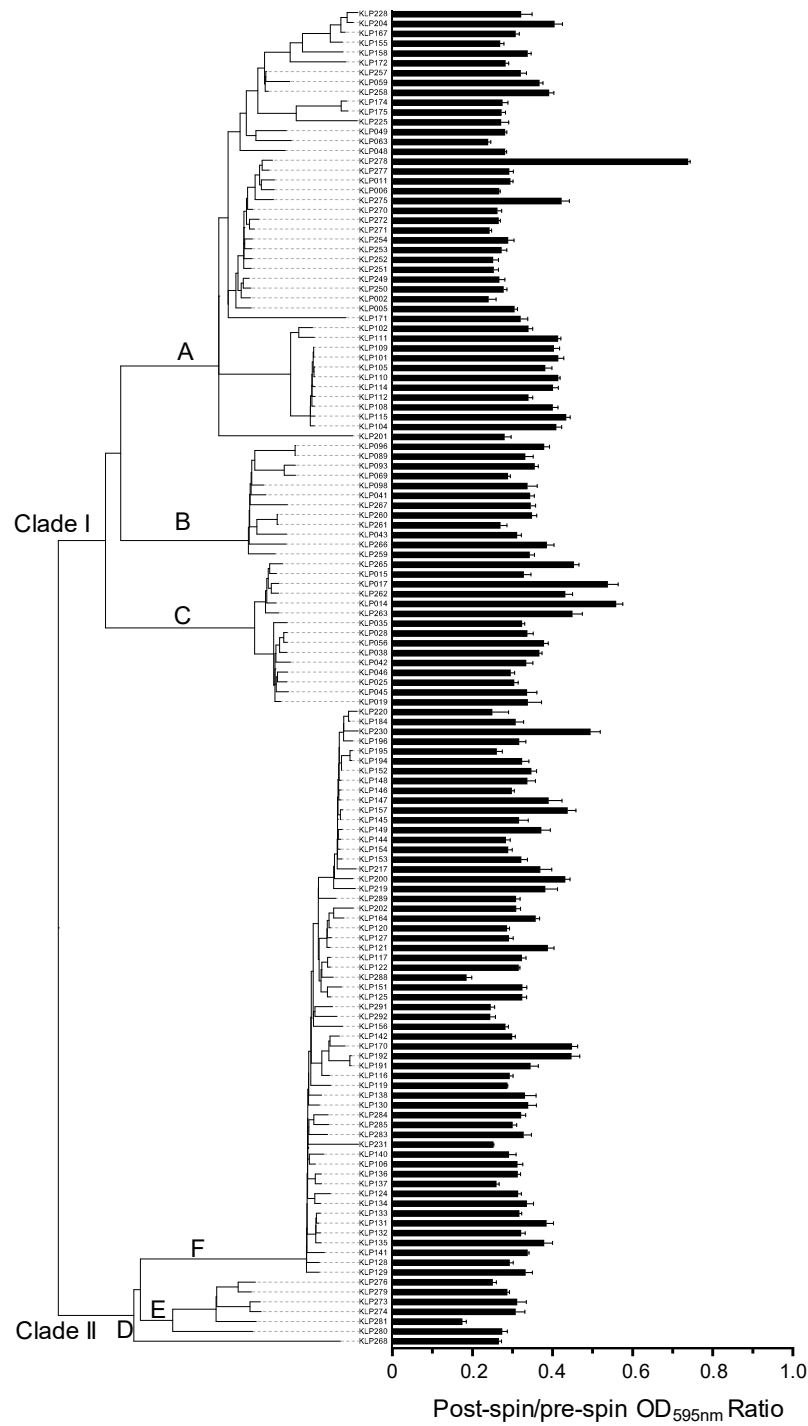

**Fig. S4B**

Supplement: FIG S4 [file mBio.01945-19-sf004.pdf]

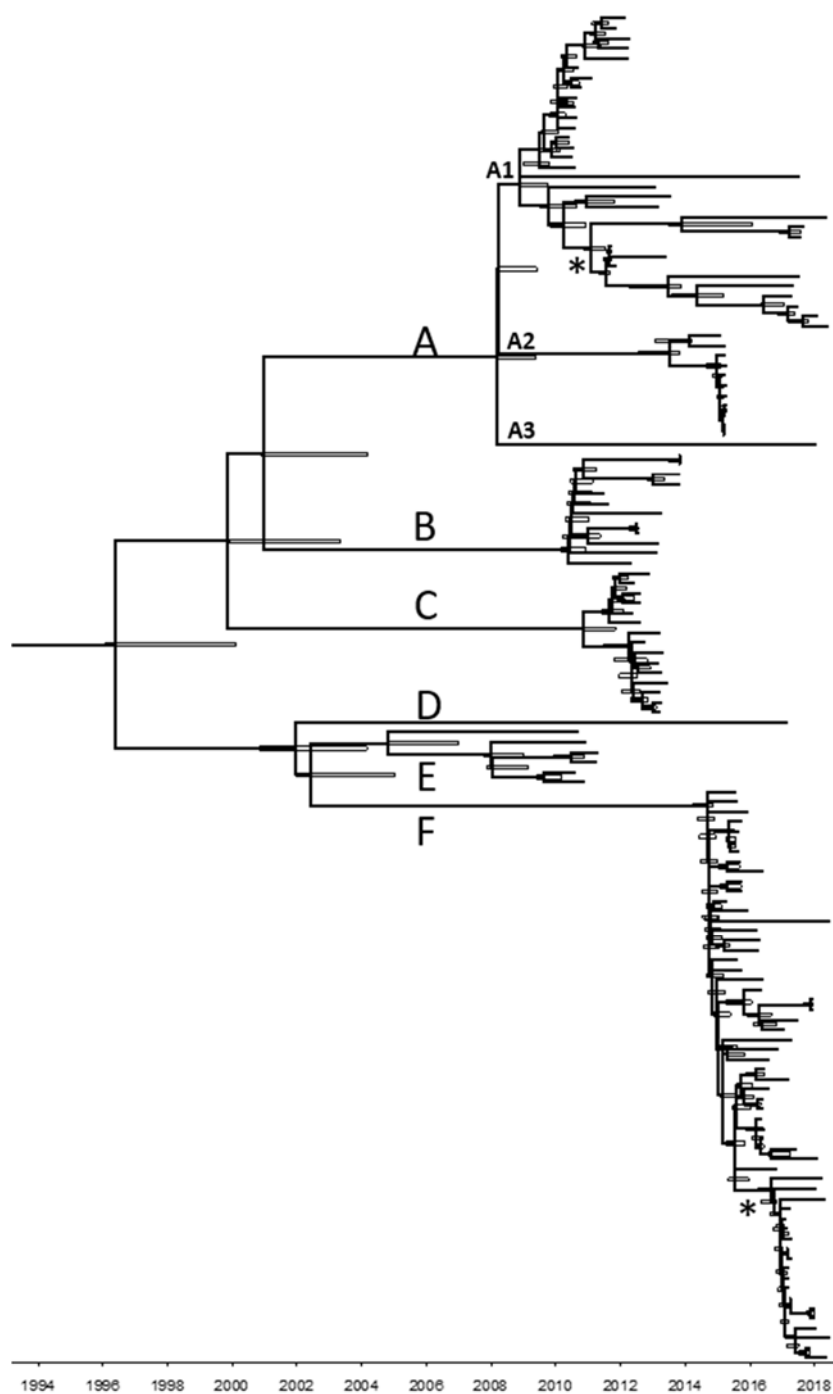

**Fig. S3**

Supplement: FIG S3 [file mBio.01945-19-sf003.pdf]

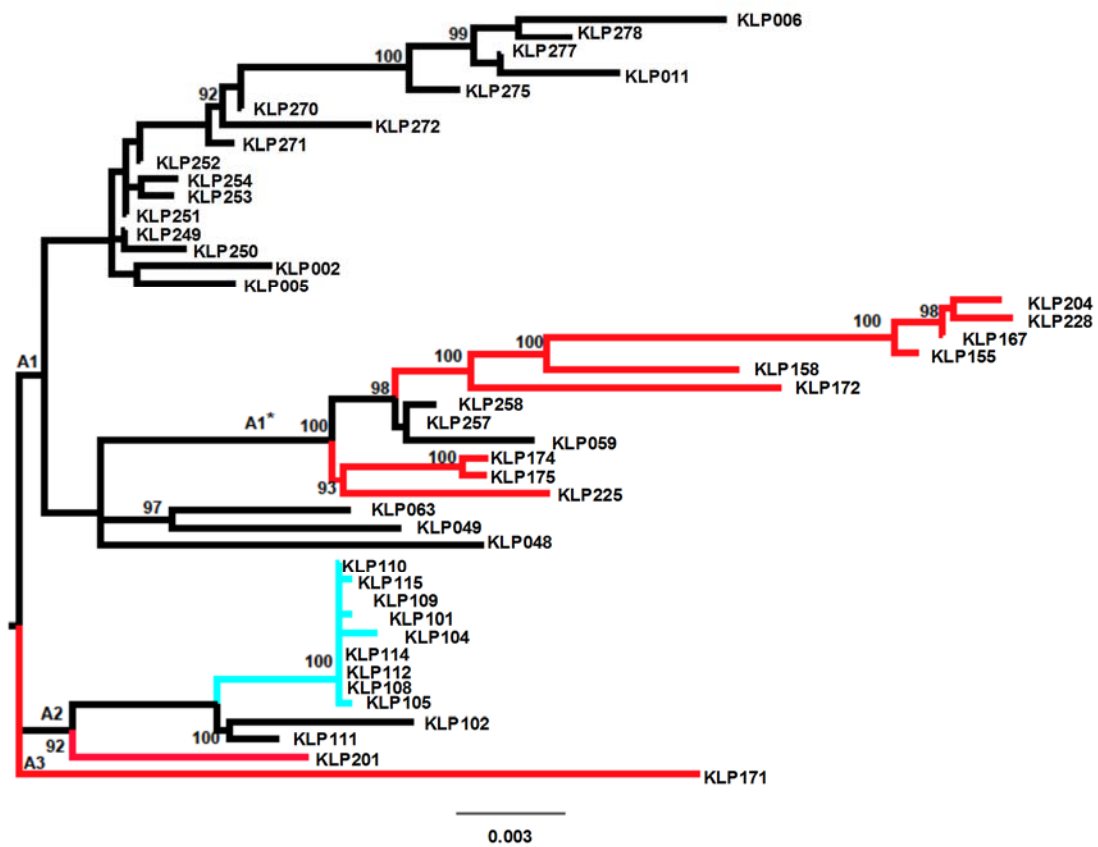

Fig. S5A

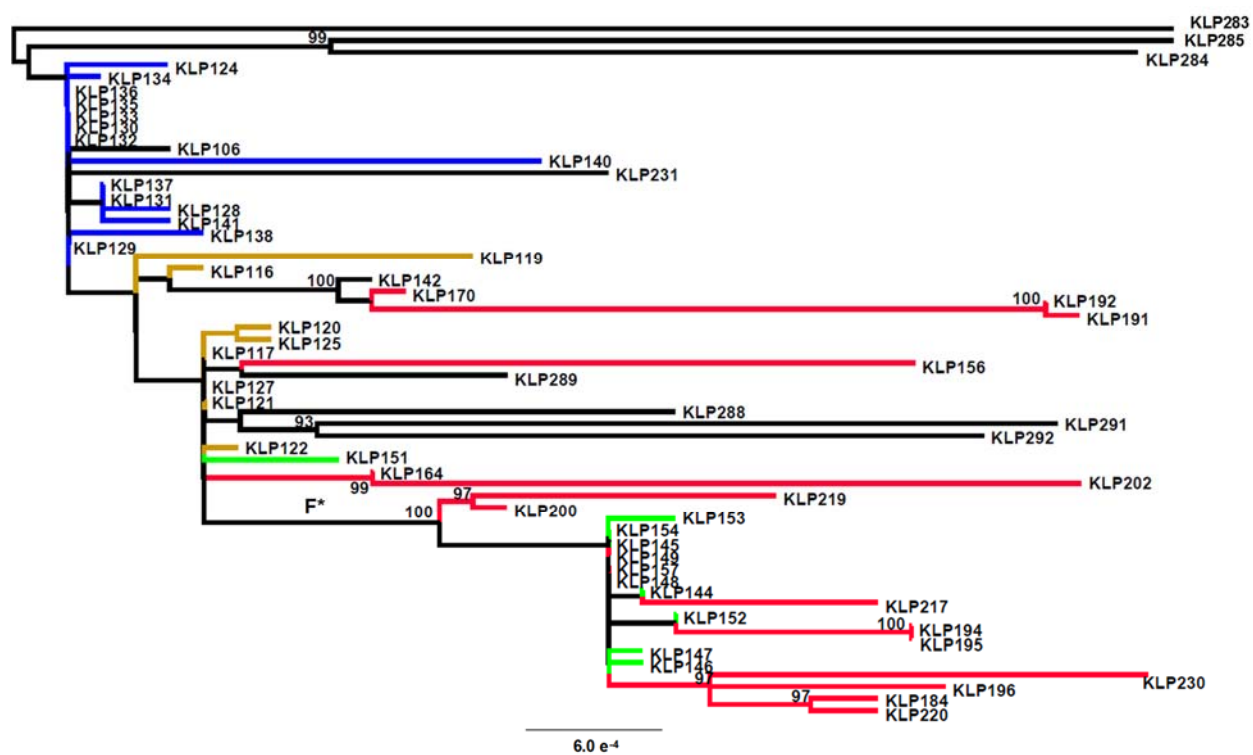

Fig. S5B

Supplement: FIG S5 [file mBio.01945-19-sf005.pdf]
